# Editorial: Biomarkers of gut blood flow, oxygenation, inflammation and NEC in neonates

**DOI:** 10.3389/fped.2023.1234832

**Published:** 2023-07-05

**Authors:** Narendra Aladangady, Ian Sanderson

**Affiliations:** ^1^ Neonatal Unit, Homerton Healthcare NHS Foundation Trust, London, United Kingdom; ^2^ Centre for Paediatrics, Blizard Institute, Queen Mary University of London, London, United Kingdom; ^3^ Centre for Immunobiology, Blizard Institute, Queen Mary University of London, London, United Kingdom

**Keywords:** necrotising enterocolitis (NEC), gut perfusion, near infrared spectroscopy, Doppler ultrasound scan, gut microbiota, preterm infant, miRNA in NEC

Editorial on the research topic Biomarkers of gut blood flow, oxygenation, inflammation and NEC in neonates

## Introduction

Necrotising enterocolitis (NEC) is a serious complication of prematurity and is known to have multifactorial aetiologies that culminate in gut inflammation and breakdown in mucosal barrier function (1, 2). Despite enormous advances in neonatal care, the global incidence of (NEC) remains stubbornly high and in some instances, it may be increasing (3) due to increased survival of babies born extremely preterm (4). The overall mortality from confirmed NEC is estimated to be around 25% (5) and rises to 50% in extreme preterm infants who require NEC surgery (4, 5). Morbidity is also common with NEC, and it is known to impact significantly on infants' long-term neurodevelopment and intestinal health (5).

A proportion of extremely preterm babies develop hypotension after birth and there is evidence that a mean arterial blood pressure of ≤30 mmHg during the first 72 h after delivery may predispose a baby to subsequent NEC (6). Anaemia is also common and may result from pulmonary or intracranial haemorrhage but primarily from phlebotomy loss associated with medical care and consequently, many babies receive blood transfusions (7). Blood transfusions significantly improve intestinal oxygenation in very preterm infants (8). However, there are uncertainties about whether hypotension, anaemia (9) or/and blood transfusion (10) produce ischaemia, reperfusion injury, and inflammation which could themselves predispose to gut injury and NEC. There is growing interest in evaluating tissue biomarkers of gut injury in neonates, particularly those representing changes in intestinal barrier integrity and permeability. There is also interest in measuring splanchnic blood flow and gut tissue oxygenation, to determine whether these could be used as biomarkers to detect or predict gut injury (1, 11).

This special topic edition series on “Biomarkers of Gut Blood Flow, Oxygenation, Inflammation and NEC in Neonates” includes four reviews and one original research article. The main objective is to review recent advances in the early prediction of NEC focussing on: tissue biomarkers; gut perfusion measurements using Near Infrared Spectroscopy (NIRS) and Doppler Ultrasound Scan (USS); alterations in microbiota in surgical NEC cases; and the potential role of micro-Ribonucleic Acid (miRNA) in NEC evaluation.

Howarth et al. review the literature for tissue biomarkers used to predict, diagnose, treat, and prognosticate NEC in preterm infants. The authors examine tissue biomarkers of hypoxic injury and inflammatory markers of gut injury, and report that neither is helpful in predicting NEC. They discuss specific gut tissue injury markers such as blood or urine fatty acid binding proteins (FABPs) and trefoil factors (TFF), stool volatile organic compounds (VOC) and stool calprotectin. New urinary biomarkers (e.g., fibrinogen peptides, Prostaglandin E2), metabolomics and proteomics are also briefly discussed. They report that FABP is the most promising for NEC prediction and diagnosis. Prediction models using a combination of clinical features together with clinical and laboratory biomarkers are explained.

In their systematic review, Howarth et al. discuss the basic principles of NIRS specifically related to gut circulation and oxygenation measurements and its current use in newborn infants. NIRS monitoring is non-invasive, safe and provides contemporaneous organ circulation/oxygenation at the neonatal cot side. The authors explain the evidence for validity of abdominal NIRS in preterm infants and its use in monitoring gut oxygenation and early detection of gut injury. The limitations of using NIRS as a potential biomarker to detect gut injury are a lack of normal measurement ranges, cost and training implications. However, NIRS could detect gut perfusion changes prior to the development of NEC and may provide an opportunity for timely interventions that improve NEC outcomes.

Murphy et al. discuss the basic principles, advantages and limitations of Doppler USS and its application in the measurement of gut blood flow in preterm infants. Superior Mesenteric Artery (SMA) Doppler USS may help in assessing the transition from fetal to newborn circulation and, the effects of feeding particularly for high-risk preterm infants. High resistance patterns of SMA flow persist after birth for approximately 24 h in infants with Fetal Growth Restriction (FGR). The high resistance patterns in SMA doppler studies suggest altered gut perfusion that may contribute to gut tissue ischaemia, dysmotility and gut injury including NEC. The evidence to recommend routine SMA doppler measurements to prevent or reduce incidence of NEC in preterm infants is lacking.

Lin et al. compare the gut microbiota in preterm infants who require surgery for NEC (≥stage 2b) to matched control infants (infants without NEC or congenital intestinal abnormality). Samples were collected at the onset of NEC and after full re-establishment of enteral feeds following NEC surgery. The authors report diversity being lower in preterm infants with NEC who required surgery (even after full recovery) when compared to controls.

Cai et al. in their systematic review, discuss the micro-Ribonucleic acid (miRNA) and its impact on the pathogenesis of NEC in preterm infants. miRNA is an endogenous non-coding single-stranded RNA that acts as a potential negative regulator. The authors discuss how miRNA influences intestinal epithelial cell barrier function and the regulation of the inflammatory process, which in turn may cause gut injury including NEC. miRNA could be used as a potential biomarker to diagnose NEC, and also for the treatment of NEC. At present, the therapeutic potential of miRNAs is still at experimental level and further clinical research is required.

## Conclusion

This special issue discusses recent advances in the measurement of gut perfusion and biomarkers of NEC in preterm infants. At present, no single biomarker seems useful in either predicting or diagnosing early NEC. Furthermore, at present, metabolomics, proteomics and miRNA are not useful in evaluating the day-to-day clinical practice. The measurement of gut NIRS is promising but will need further clinical evaluation.
